# TSPO, a Mitochondrial Outer Membrane Protein, Controls Ethanol-Related Behaviors in *Drosophila*

**DOI:** 10.1371/journal.pgen.1005366

**Published:** 2015-08-04

**Authors:** Ran Lin, Danielle Rittenhouse, Katelyn Sweeney, Prasanth Potluri, Douglas C. Wallace

**Affiliations:** 1 Center for Mitochondrial and Epigenomic Medicine, Children’s Hospital of Philadelphia Research Institute, Philadelphia, Pennsylvania, United States of America; 2 Department of Pathology and Laboratory Medicine, Perelman School of Medicine, University of Pennsylvania, Philadelphia, Pennsylvania, United States of America; Stanford University School of Medicine, UNITED STATES

## Abstract

The heavy consumption of ethanol can lead to alcohol use disorders (AUDs) which impact patients, their families, and societies. Yet the genetic and physiological factors that predispose humans to AUDs remain unclear. One hypothesis is that alterations in mitochondrial function modulate neuronal sensitivity to ethanol exposure. Using *Drosophila* genetics we report that inactivation of the mitochondrial outer membrane translocator protein 18kDa (TSPO), also known as the peripheral benzodiazepine receptor, affects ethanol sedation and tolerance in male flies. Knockdown of dTSPO in adult male neurons results in increased sensitivity to ethanol sedation, and this effect requires the dTSPO depletion-mediated increase in reactive oxygen species (ROS) production and inhibition of caspase activity in fly heads. Systemic loss of dTSPO in male flies blocks the development of tolerance to repeated ethanol exposures, an effect that is not seen when dTSPO is only inactivated in neurons. Female flies are naturally more sensitive to ethanol than males, and female fly heads have strikingly lower levels of dTSPO mRNA than males. Hence, mitochondrial TSPO function plays an important role in ethanol sensitivity and tolerance. Since a large array of benzodiazepine analogues have been developed that interact with the peripheral benzodiazepine receptor, the mitochondrial TSPO might provide an important new target for treating AUDs.

## Author Summary

Alcohol use disorders (AUDs) affect millions of patients worldwide and result in high social and economic burdens. Although environmental factors are involved, there are clear genetic components to AUDs. Both the acute sedating effect of alcohol exposure and alcohol tolerance contribute to long term risk for alcohol dependence and addiction. Yet the genetic etiology of AUDs remains to be determined. The mitochondria play a central role in ethanol metabolism and are important in many aspects of cellular physiology such as REDOX and ROS regulation, and apoptosis. The mitochondrial outer membrane translocator protein 18 kDa (TSPO) binds the benzodiazepines and perhaps other addictive drugs, and thus may play a role in AUDs. Since *Drosophila* is a well-established model for ethanol-related behaviors, we have developed systems for manipulating the *Drosophila tspo* gene and protein. With these systems, we have discovered that neuronal TSPO controls sensitivity to ethanol sedation via ROS and caspase-mediated signaling and that systemic TSPO levels are important in the development of tolerance to repeated ethanol exposure. Given the variety of known TSPO ligands, and the common mechanisms of various abusive substances, our studies suggest that TSPO might be a promising target to combat alcoholism as well as addiction to other drugs.

## Introduction

Alcohol is one of the most widely used drugs worldwide, but long term consumption leads to its abuse and dependence. An estimated 17.6 million adults in the United States have AUDs with associated health concerns of alcohol dependence, liver cirrhosis, cancer, and injuries. From 2006 through 2010, this generated an annual average of about 88,000 alcohol-related deaths and 2.5 million years of potential life lost [1,2].

To develop therapeutic strategies for alcoholism it will be necessary to determine the molecular and cellular mechanisms underlying AUDs. Considerable effort has been invested in determining the role of the central nervous system in the etiology of AUD [3–5] but many features of the AUDs remain unexplained.

Neuronal function is highly dependent on mitochondrial bioenergetics [6,7]. In addition to the direct metabolizing of ethanol, the mitochondria are central to a wide range of essential neuronal cell functions including ATP synthesis, ROS production and REDOX homeostasis, Ca2+ buffering, and the metabolic regulation of apoptosis [8–10]. In humans mitochondrial DNA (mtDNA) alterations have been correlated with alcoholism, involving both acute ethanol responses and chronic damage [11–16]. In rodents, hepatic mtDNA depletion is seen in alcohol exposed mice [17] and mtDNA complex I gene variants have been correlated with “non-drinker” versus “drinker” rat lines derived from the same founder strain [18]. Variation in the mtDNA genes have also been shown to have profound effects of nuclear gene expression [19].

In previous studies we showed that the nuclear DNA coded *Drosophila* translocator protein 18kDa (dTSPO, CG2789) is localized in outer mitochondrial membrane (OMM) and important for regulating mitochondria bioenergetics, ROS production, caspase activity, and apoptotic function [20]. In humans, TSPO ligands are widely used in neuroimaging for neurodegenerative diseases and neuronal injuries, both of which are associated with increased brain TSPO levels and distribution [21]. As the previous nomenclature (peripheral benzodiazepine receptor, PBR) implies, TSPO binds the benzodiazepines and other psychotrophic drugs associated with tolerance and addiction [22]. Thus we hypothesized the TSPO may be an important factor in addiction to ethanol.

*Drosophila’s* sensitivity and tolerance to ethanol are similar to humans and rodents. Ethanol results in biphasic locomotor alterations. At lower doses ethanol acts as a stimulant, but at higher doses it acts as a depressant [23]. After repeated alcohol stimulation, tolerance is developed, defined as acquired resistance. Tolerance is thought to be an intermediate step to alcohol dependence and addiction [24].

Here we report that in male *Drosophila*, neuronal inactivation of dTSPO sensitizes flies to ethanol sedation, mediated by increased ROS production and decreased caspase activation. Furthermore, systemic but not neuronal loss of dTSPO inhibits the development of tolerance. By contrast, females are constitutively more sensitive to ethanol sedation than males and they have much lower dTSPO mRNA in their brains. Therefore, the mitochondrial TSPO is an important mediator of ethanol sensitivity and tolerance and contributes to gender-specific differences in alcohol sensitivity.

## Results

### Neuronal depletion of dTSPO increases ethanol sensitivity in adult male flies

**Fig 1:**
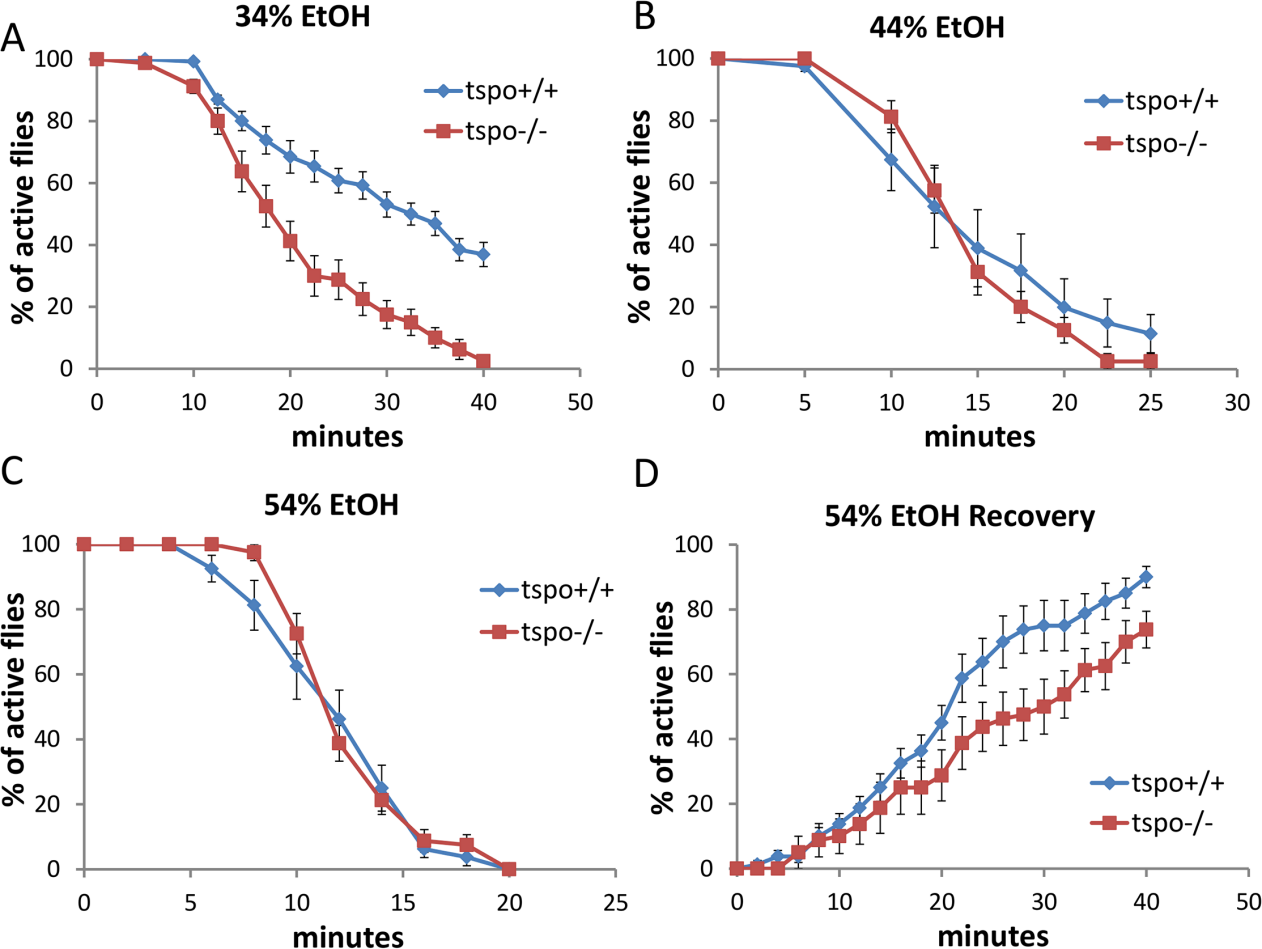
Increased ethanol sensitivity in male *tspo* mutant flies. Sensitivity to acute ethanol sedation was increased in *tspo*-/- flies compared with *tspo*+/+ flies (A-C). (A) With 34% ethanol solution, half sedation time for *tspo*+/+ was 30.6±1.9 min and for *tspo*-/- was 17.8±1.5 min, p = 0.01, n = 13 vials tested. (B) With 44% ethanol solution, half sedation time for *tspo*+/+ was 14.3±2.2 min and for *tspo*-/- was 13.8±0.6 min, p > 0.05, n = 8 vials tested. (C) With 54% ethanol solution, half sedation time for *tspo*+/+ was 11.3±0.8 min and for *tspo*-/- was 11.3±0.3 min, p > 0.05, n = 8. (D). The rate for recovery after ethanol withdraw was slower in *tspo*-/- than *tspo*+/+ flies, half recovery time for *tspo*+/+ was 20.4±2.1 min and for *tspo*-/- was 28.1±3.6 min, p > 0.05, n = 8. Data presented as mean ± SEM.

Acute ethanol sensitivity was analyzed by placing flies in vials closed by cotton clogs soaked with varying concentrations of ethanol thus exposing the flies to ethanol vapor. During initial exposure the flies flew to the top of the vial, and exhibited hyperactivity for a few minutes. With longer exposure, the flies became sedated and remained at bottom of the vial without locomotion. Wild type flies became comparably sedated whether the ethanol-soaked clogs were at the top of the vials or the vials were inverted with clogs at the bottom (S1 Fig). Moreover, the *Drosophila* showed a dose-dependent response to ethanol using this protocol (Fig 1). Therefore, the ethanol effects observed in the following experiments were due to the ethanol concentration rather than an environmental factor such as hypoxia due to ethanol vapor exclusion of air.

The *tspo*[EY00814] mutant *Drosophila* has a P-element inserted into the *tspo* gene leading to loss of dTSPO expression [20]. Male *tspo*-/- flies exhibited higher sedation sensitivity than *tspo* +/+ flies when exposed to ethanol vapor from 34% ethanol solution (Fig 1A) while at 44% or 54% ethanol vapor both the *tspo*-/- and *tspo* +/+ flies exhibited the same sensitivity (Fig 1B and 1C). Post sedation, we tested for the recovery from ethanol sedation by replacing the ethanol-soaked clogs with normal clogs. This revealed that at 54% ethanol exposure *tspo*-/- males were slower to recover than the *tspo* +/+ male flies (Fig 1D). Thus, *tspo*-/- male flies are more sensitive to ethanol sedation than their *tspo* +/+ counterparts. While the *rempA* gene overlaps with the *tspo* gene, *rempA*-/- deficiency is not the cause of the ethanol phenotypes since *rempA*-/- flies exhibit comparable sensitivity to 34% ethanol vapor as wild type flies (S2 Fig).

Since the *tspo* mutation is present in all developmental stages of the fly, it could act through creating a developmental abnormality. However, Hematoxylin-Eosin histological comparison of the brains of male tspo-/- and +/+ flies did not reveal any gross anatomical differences (S3 Fig).

**Fig 2:**
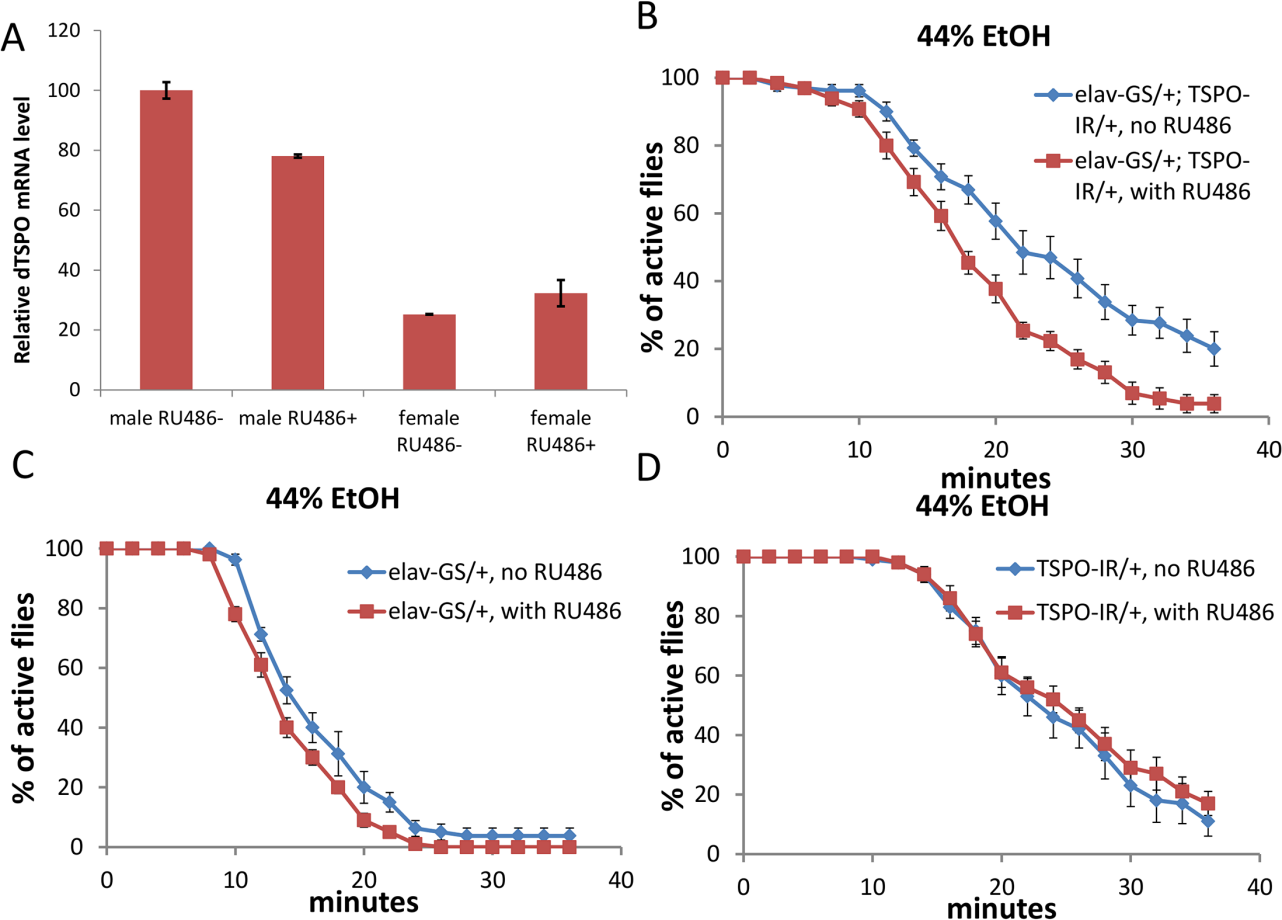
High male brain expression of TSPO associated with increased ethanol sensitivity in male neuronal dTSPO knockdown flies. (A) Levels of dTSPO mRNA in the heads of elav-GS/+; TSPO-IR/+ male and female flies with or without RU486 dsRNA induction, n = 3 groups of flies tested. Data presented as mean ± SEM. *** p < 0.001. (B-D) Gene switch and control flies with or without RU486 (elav-GS/+ = elav-GeneSwitch and TSPO-IR/+ = UAS-dTSPO-RNAi). To induce gene switch, the flies were raised on regular food with 50 μl of 4 mg/ml RU486 added on the surface of the food in vials for three days. (B) Sensitivity of elav-GS/+;TSPO-IR/+ flies to 44% ethanol vapor with and without RU486, half sedation time with RU486 was 16.0±0.6 min and without RU486 was 23.3±1.5, p < 0.001, n = 10. (C) Sensitivity of flies harboring elav-GS/+ with or without RU486 exposed to 44% ethanol vapor, half sedation time with RU486 was 14.0±0.5 min and without RU486 was 15.3±0.9, p > 0.05, n = 13, vials tested. (D) Sensitivity of flies harboring only TSPO-IR/+ exposed to 44% ethanol and with and without RU486, half sedation time with RU486 was 25.0±2.0 min and without RU486 was 22.5±1.0, p > 0.05, n = 10.

To determine whether the increased ethanol sensitivity was attributable to dTSPO function in neurons, we depleted dTSPO in neurons by inducing dsRNA (RNAi) to knockdown dTSPO mRNA in adult flies following eclosion (days after eclosion, dae). We used the Gal4-GeneSwitch/UAS system [25] in which Gal4 is activated within the flies when fed with mifepristone (RU486). The activated Gal4 binds to the UAS of the UAS-dTSPO-RNAi which induces the dsRNA expression and inhibition of the dTSPO mRNA. Since the Gal4 element is expressed under the neuronal specific elav promoter (elav-GeneSwitch), this switch was restricted to neurons. In this way, the flies were permitted to progress through larval and pupal development with normal TSPO activity, and following eclosion, the dTSPO RNAi was induced in neurons by exposure to RU486. Male flies harboring both elav-GeneSwitch and UAS-dTSPO-RNAi (elav-GS/+; TSPO-IR/+)(GS means ‘Gene Switch’ and IR means ‘Inverted Repeats’) cassettes that were exposed to RU486 post eclosion had reduced head dTSPO mRNA as quantified by RT-PCR (Fig 2A). Therefore, activation of the elav-GS/+; TSPO-IR/+ system with RU486 specifically depletes dTSPO mRNAs in the neurons.

In parallel with the whole body knockouts, the elav-GS/+; TSPO-IR/+ RU486 knockdown male flies exhibited faster ethanol sedation in the presence of 44% ethanol vapor than did flies who were not exposed to RU486 (Fig 2B). RU486 exposure of flies harboring only the neuronal elav-GeneSwitch (elav-GS/+) or the UAS-dTSPO-RNAi (TSPO-IR/+) cassette had no effect on the ethanol sensitivity (Fig 2C and 2D). Similarly, elav-GS/+; TSPO-IR/+ male flies exposure to 34% ethanol also showed increase sedation after RU486 induction relative to uninduced flies (S4A Fig). After 55% ethanol sedation (S4B Fig), the RU486-induced flies were slower to recover (S4C Fig). The difference between the RU486 induced and uninduced flies was not due to differential alcohol absorption or metabolism since after a brief exposure to 44% ethanol vapor both groups of fly heads (with and without RU486) had the same ethanol concentration (S4D Fig). Hence, dTSPO inactivation in adult neurons is sufficient to sensitize male flies to ethanol exposure.

**Fig 3:**
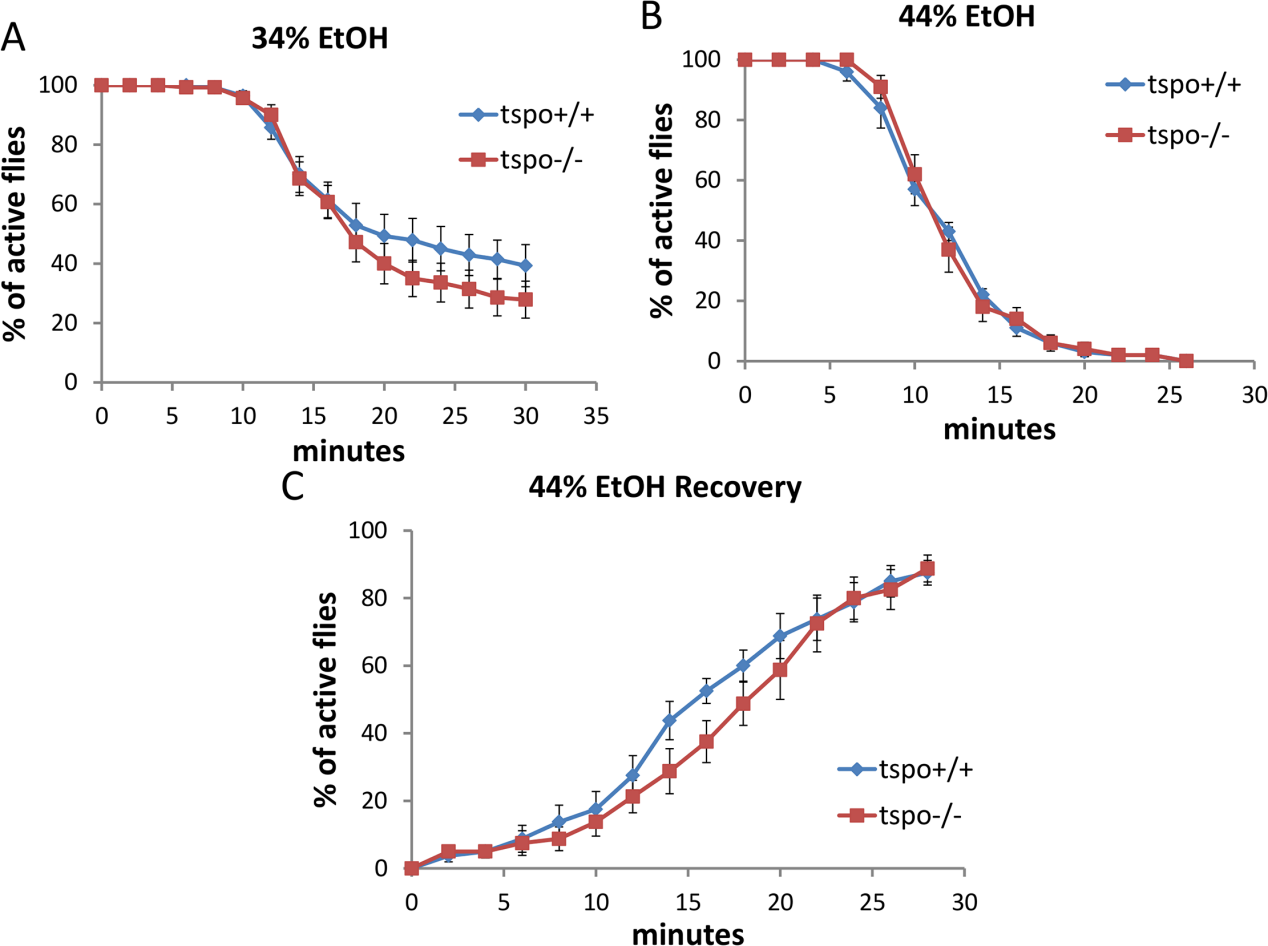
Comparable ethanol sensitivity in female *tspo*-/- and *tspo* +/+ flies. (A) With 34% ethanol vapor, half sedation time for *tspo*+/+ was 23.0±2.5 min and for *tspo*-/- was 20.0±1.7 min, p > 0.05, n = 14 vials tested. (B) With 44% ethanol vapor half sedation time for *tspo*+/+ was 11.0±0.5 min and for *tspo*-/- was 11.0±0.5 min, p > 0.05, n = 10. (C) Recovery after withdraw from exposure to 44% ethanol vapor, half recovery rate for *tspo*+/+ was 16.0±1.2 min and for *tspo*-/- was 18.0±1.2 min, p > 0.05, n = 8. Data presented as mean ± SEM.

Male and female flies exhibit sexual dimorphic response to ethanol exposure [4] and this sexual dimorphism was also observed in the brains of the TSPO knockout and knockdown flies. Male *tspo*-/- flies showed an increased sensitivity to ethanol sedation relative to *tspo* +/+ flies with 34% ethanol exposure and delayed recovery from 54% sedation (Fig 1A and 1D) while female *tspo*-/- and *tspo* +/+ flies showed no difference in their response to 34% ethanol exposure (Fig 3A–3C).

In elav-GS/+; TSPO-IR/+ female flies, after neuronal inactivation of dTSPO by dsRNA expression, there was no effect on the sedation rate with exposure to 34% or 44% ethanol solution (S5A and S5B Fig). Furthermore, only slightly delayed recovery was seen for female flies after 44% vapor sedation (S5C Fig). The marked difference between male and female elav-GS/+; TSPO-IR/+ flies’ sensitivity to ethanol following dTSPO inactivation by RU486 induction correlated with male fly heads having about four times the level of dTSPO mRNA as female heads. Moreover, neuronal knockdown of dTSPO reduced male head TSPO mRNA level but had no effect on female head TSPO mRNA level (Fig 2A). Therefore, the lack of sensitivity of female flies to neuronal inactivation of dTSPO is likely do to a gender-specific lack of TSPO in female fly brains.

### Increased ROS mediated sensitivity to ethanol in dTSPO-depleting flies

**Fig 4:**
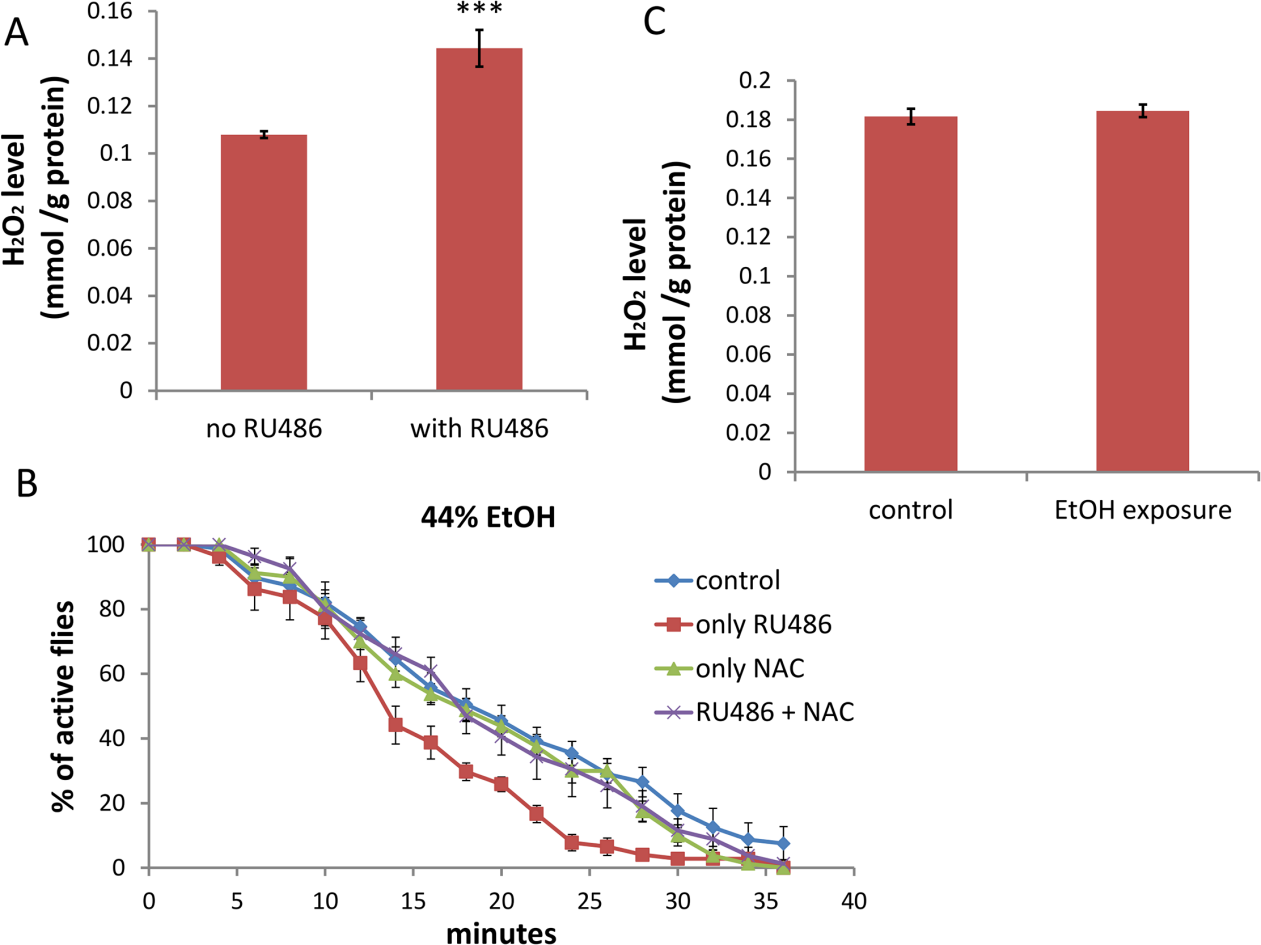
Depletion of dTSPO in neurons increases ROS which is necessary to sensitize flies to ethanol sedation. (A) H_2_O_2_ content was higher in heads of elav-GS/+; TSPO-IR/+ flies with RU486 induction of dTSPO dsRNA, n = 8 groups of flies tested. (B) Inhibition of increased sedation sensitivity to 44% ethanol vapor of elav-GS/+; TSPO-IR/+ flies treated with RU486 when treated together with the antioxidant N-Acetyl-L-Cysteine (NAC) (red versus blue curve). To induce gene switch, the flies were raised on regular food with 50 μl of 4 mg/ml RU486 added on the surface of the food in vials for three days. To inhibit ROS production, 20 μl of 500 mM NAC was premixed with the 50 μl RU486 solution and the mixture added to the surface of the food in vials for five days. Half sedation time for control feeding flies was 17.6±1.3 min, for RU486-only feeding flies was 12.9±1.2 min, for NAC-only feeding flies was 15.9±1.5 min, for RU486 and NAC feeding flies was 17.6±1.6 min. Control versus RU486-only flies, p = 0.0182; NAC versus NAC+RU486 flies, p = 0.4292, n = 8, vials tested. (C) H_2_O_2_ content of heads of male wild type flies after 20 minutes exposure to 44% ethanol vapor (n = 3 groups of flies tested). Data presented as mean ± SEM. *** p < 0.001.

To determine what might be the physiological basis of the neuron-specific effects of dTSPO deficiency on male ethanol sedation, we examined the effects on ROS production, which we previously found was increased in dTSPO deficient mitochondria [20]. ROS has been identified as modulator of neuronal activity [26]. Using Amplex Ultrared to determine the amount of H_2_O_2_ in fly heads, we found that H_2_O_2_ levels were higher in male elav-GS/+; TSPO-IR/+ flies treated with RU486 than untreated flies (Fig 4A). Hence, neuronal dTSPO knockdown increased fly head H_2_O_2_ production. When these flies were fed with N-Acetyl-L-Cysteine (NAC), an efficient antioxidant, the enhanced sedation effect of the dTSPO knockdown flies to 44% ethanol vapor was negated (Fig 4B). In *tspo* +/+ male flies, exposure of 44% ethanol vapor for 20 minutes resulted in sedation of most of the flies but did not significantly alter H_2_O_2_ content in fly heads (Fig 4C). Hence, the increase in ROS is not caused by ethanol exposure. Rather, dTSPO inactivation in neurons up-regulates ROS and the increased ROS is responsible for the enhanced ethanol sensitivity of the dTSPO-depleted flies.

### Aging-associated sensitivity to ethanol sedation is attenuated in dTSPO-depleting flies

Since ethanol sedation sensitivity was controlled by TSPO and TSPO expression declines in *tspo* +/+ flies to a minimum at 30 dae (S5B Fig of [20]), we determined whether ethanol sensitivity changes during aging. Male *tspo* +/+ and *tspo*-/- flies were tested with 44% and 54% ethanol sedation at different ages i.e. young (about 5 dae), mid-age (about 20 dae), and old (about 35 dae). Wild type (*tspo*+/+) flies exhibited increased sedation as they aged, with the effect already evident by 20 dae. *tspo*-/- flies also displayed and increased predilection to sedation with age (S6C Fig), but they were initially significantly more sensitive to ethanol. This is consistent with their higher level of oxidative stress as demonstrated by the marked reduction in their ROS-sensitive mitochondrial aconitase activity (Fig 6 of [20]).

### Neuronal dTSPO depletion suppresses caspase activity which is sufficient to produce ethanol sensitivity

**Fig 5:**
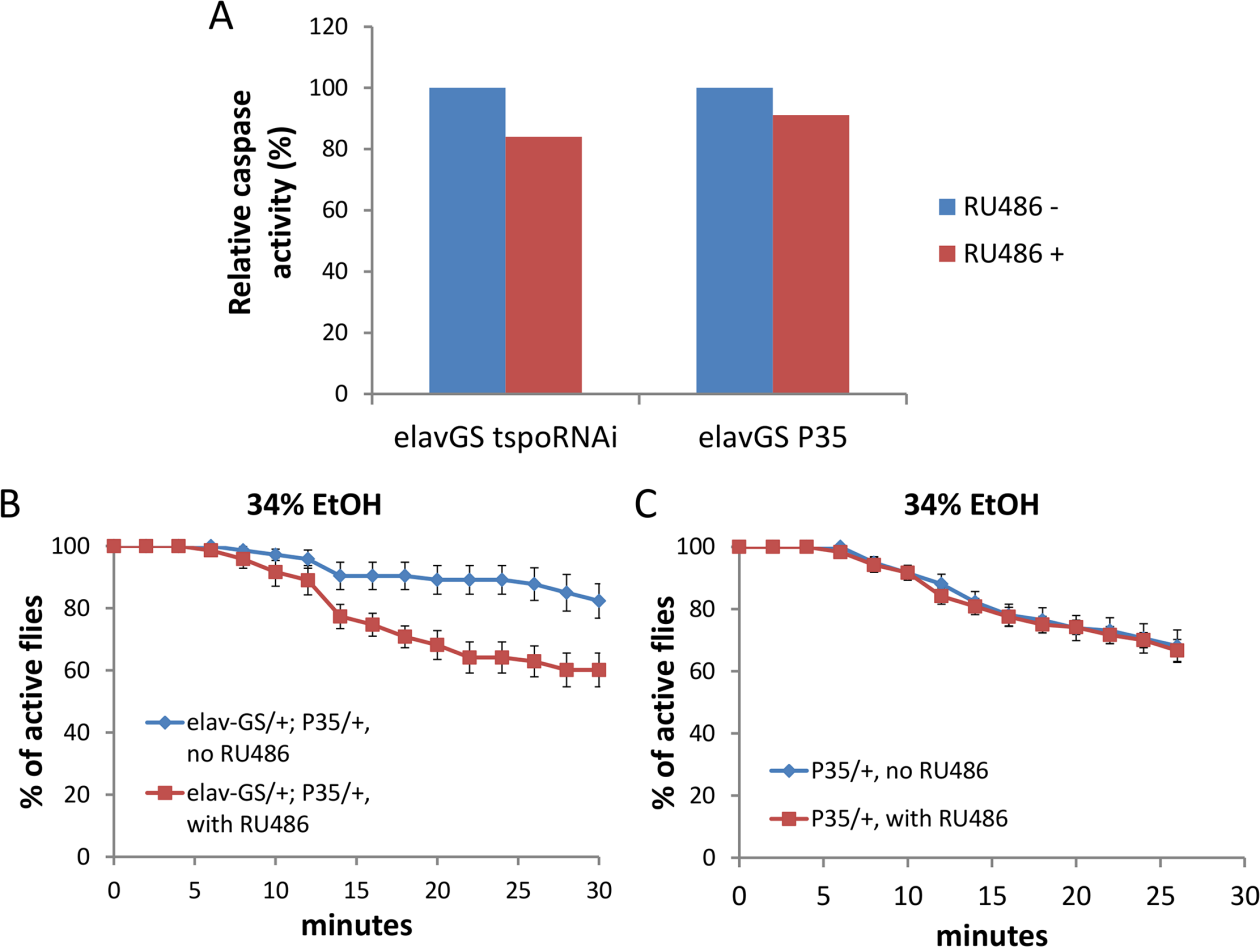
Depletion of dTSPO in neurons suppresses caspase activity which is sufficient to increase ethanol sensitivity. Gene switch was accomplished as in Fig 2. (A) Caspase 3/7 activity was moderately reduced in heads of elav-GS/+; TSPO-IR/+ or elav-GS/+; p35/+ (elav-GeneSwitch plus UAS-p35) flies when the dTSPO dsRNA or p35 were induced with RU486. All groups were measured twice, and data presented as mean. Since the TSPO-IR and P53 products are only expressed in neurons, but the caspase activity was assayed in whole heads, the ~20% decrease in caspase 3/7 underrepresents the extent of caspase reduction in neurons. This is demonstrated by whole body knockout of TSPO in which the relative whole body caspase 3/7 activity of *tspo* +/+ flies was 1.000±0.008 and of *tspo*-/- flies was 0.078±0.015, p < 0.001, n = 4. (B) Induction of caspase inhibitor p35 with RU486 significantly increased sensitivity to 34% ethanol vapor, Chi Square log rank test, p = 0.0006, n = 8 vials tested. (C) Flies harboring only UAS-p35 (p35/+) exposed to 34% ethanol vapor with or without RU486 were not different, Chi Square log rank test, p = 0.846, n = 12. Data presented as mean ± SEM.

Depletion of dTSPO in flies suppresses caspase activation and impedes apoptosis [20]. However, caspase also has cell death-independent functions which might be involved in neuronal control [27]. The activity of caspase 3/7, the most downstream caspase in the intrinsic apoptosis pathway, was decreased in heads of flies with dTSPO-depleted neurons (Fig 5A). Neuronal expression of the caspase inhibitor protein, p35, also reduced caspase 3/7 activity to a similar degree as induction of the TSPO dsRNA (Fig 5A). The level of caspase reduction in TSPO knockdown and p35 induced neurons is likely to be much greater than shown in Fig 5A where whole brain homogenates were assayed. Whole brain homogenates mix the enzymes of all cell types most of which are not neurons and thus not subject to dTSPO knockdown. Supporting this speculation, caspase 3/7 activity of whole body homogenate *tspo*-/- flies was tenfold lower than that of *tspo* +/+ flies (Fig 5, legend). Neuronal expression of the caspase inhibitor protein, p35, also increased sensitivity of male flies to ethanol sedation when exposed to 34% ethanol vapor (Fig 5B and 5C). This phenocopyied the dTSPO knockdown flies and confirmed the importance of reduced neuronal caspase 3/7 in ethanol sensitivity. Hence, both increased ROS production and decreased caspase activity in neurons are important in enhanced ethanol sensitivity.

### Systemic but not neuronal depletion of dTSPO prevents flies from ethanol tolerance

**Fig 6:**
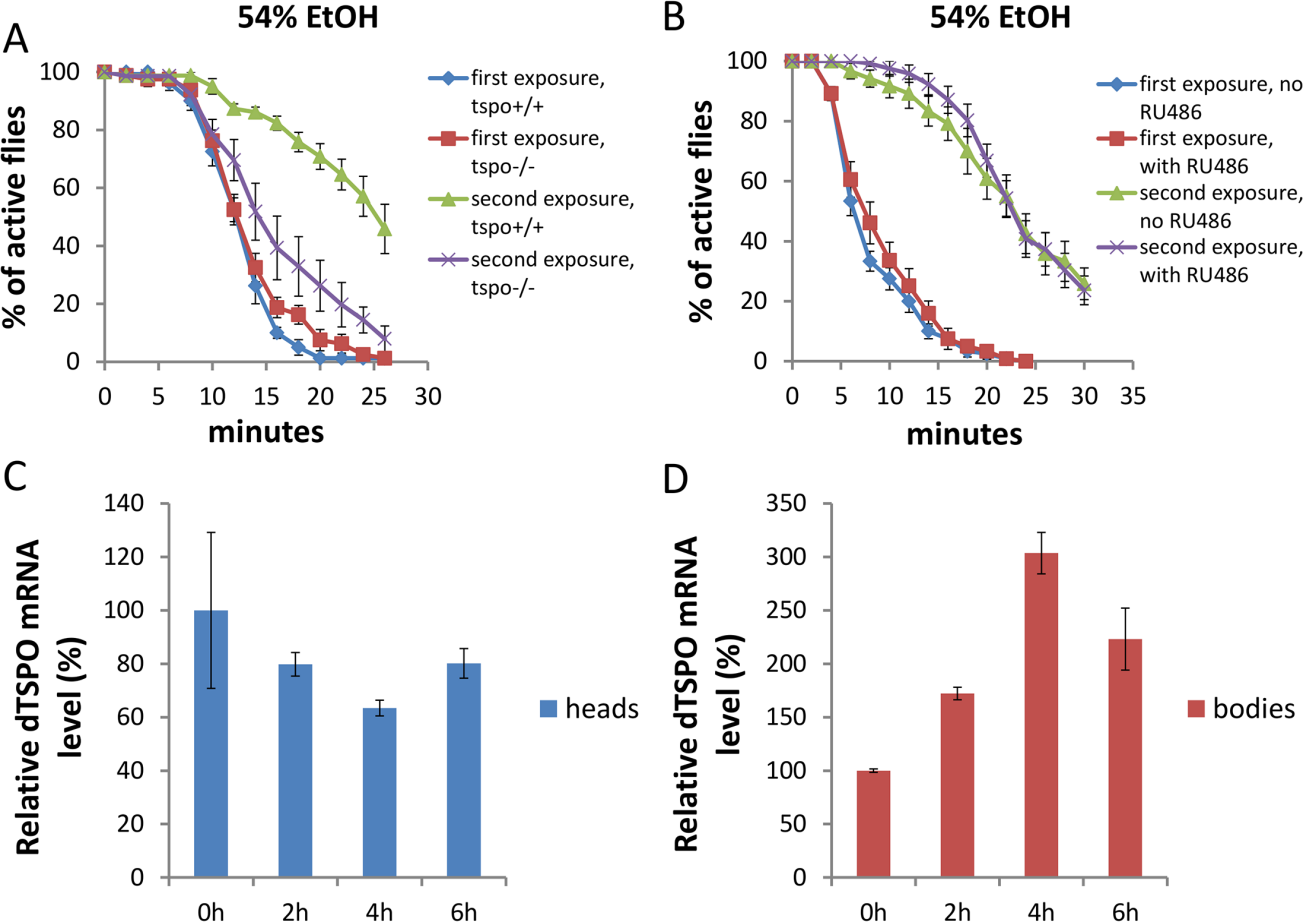
Systemic loss of dTSPO inhibits the development of ethanol tolerance. Tolerance is revealed as a longer period required for sedation at a second exposure to 54% ethanol solution vapor. (A) Differential sedation from first versus second ethanol exposure of *tspo* +/+ versus *tspo*-/- flies: For *tspo* +/+ flies half sedation time for first exposure was 12.0±0.5 and for second exposure was 26.4±1.9, p < 0.001. For *tspo*-/- flies half sedation time for first exposure was 12.3±0.3 and for second exposure was 15.0±1.6, p = 0.11. Difference between second exposure sedation of *tspo* +/+ and *tspo*-/- flies, p < 0.001, n = 8, vials tested. (B) Differential sedation from first versus second exposure of elav-GS/+; TSPO-IR/+ flies with or without RU486 induction, n = 12. Gene switch was induced as in Fig 2. (C) dTSPO mRNA levels in the heads of tspo+/+ male flies after exposure to 54% ethanol vapor showing progressive loss of dTSPO mRNA for the first 4 hours after exposure followed by partial recover to original levels, n = 3 groups of flies tested. (D) dTSPO mRNA levels in the bodies of male *tspo*+/+ flies after exposure to 54% ethanol vapor showing a progressive increase up to 4 hours after exposure followed by a decline (n = 3). Data presented as mean ± SEM.

To investigate the development of ethanol tolerance (reduced ethanol sensitivity following repeated ethanol exposures), we exposed flies to ethanol, allowed them to recover for 6 hours, and then exposed the flies to the same ethanol concentration again and monitored their sedation. In *tspo* +/+ male flies, the sedation for second exposure to 54% ethanol solution vapor was significantly delayed compared with first exposure (Fig 6A), indicating tolerance formation. However, *tspo*-/- male flies exhibited no diminished sedation sensitivity between the first and second ethanol exposure (Fig 6A). Hence, the systemic inactivation of dTSPO prevented male flies from developing tolerance. In contrast to male flies, female *tspo*-/- flies developed ethanol tolerance similar to *tspo* +/+ flies (S7 Fig). Hence, loss of ethanol tolerance in *tspo*-/- flies is also gender-specific.

To determine if the effect of dTSPO on tolerance is attributable to neurons, we compared elav-GS/+; TSPO-IR/+ flies with or without RU486 to induce TSPO dsRNA. Knockdown of dTSPO in adult male neurons had no effect on the development of tolerance following a second ethanol exposure to 54% ethanol vapor (Fig 6B). Hence the suppression of tolerance in dTSPO-depleting flies was not driven by neuronal dTSPO levels.

Since there might be other cell types in which dTSPO functions in tolerance formation, we isolated the heads and bodies of male *tspo* +/+ flies to examine the expression of dTSPO during tolerance. Within 4 hours after first exposure to 54% ethanol vapor, the amount of dTSPO mRNA in heads was decreased while the dTSPO mRNA in bodies was markedly increased. Both head and body dTSPO mRNA levels began to normalize at 6 hours post exposure (Fig 6C and 6D). Therefore, tolerance is associated with the induction of dTSPO in fly bodies, which is consistent with the loss of the capacity to develop tolerance in *tspo*-/- flies but not in elav-GS/+; TSPO-IR/+ induced flies.

## Discussion

We have found that TSPO is a mitochondrial modulator of ethanol sensitivity and tolerance in *Drosophila*. Inactivation of dTSPO in either the whole body or in adult fly neurons conferred increased sensitivity of males but not females to ethanol exposure. The increased ethanol sensitivity associated with dTSPO deficiency is a product of increased ROS production and decreased caspase activity in neurons. However, inhibition of the development of ethanol tolerance was related to systemic but not neuronal TSPO levels and this correlated with the induction of TSPO mRNA in fly bodies on ethanol exposure. Hence, our results show that TSPO is an essential mediator of alcohol sensitivity and tolerance, though not involving all the same tissue types.

### Involvement of ROS and caspase in dTSPO-modulated ethanol sensitivity

The involvement of TSPO-mediated increased neuronal ROS production and decreased caspase activity in the sensitivity to ethanol sedation is consistent with reports that oxidative stress and caspase-mediated apoptosis contribute to brain pathology [28]. Since TSPO controls mitochondrial ROS production and caspase activation [20], it follows that modulation of ROS levels and caspase activity could mediate ethanol sensitivity.

Inactivation of *tspo* increased ROS production and NAC negated the enhanced sensitivity to ethanol demonstrating that increased neuronal ROS is related to increased ethanol sedation sensitivity. Given the short exposure period of the flies to ethanol, the ROS effect is most likely due to its second messenger action [26] rather than due to a cell death mechanism. This is consistent with the recent report showing that expression of oxidative stress genes can be altered by ethanol exposure and their functions are essential for ethanol sensitivity [29,30]. It is possible that TSPO-deficiency induced ROS production could also participate in development of tolerance, but this effect must be mediated by cells other than neurons.

Inactivation of dTSPO also inhibits caspase activity[20] and inhibition of neuronal caspase activity also sensitized flies to ethanol sedation. This was confirmed by expression of the caspase inhibitor p35 resulting in increased ethanol sensitivity. Since caspase has been shown to function in neuronal apoptosis-independent pathways to control neuronal activity in both developmental and adult stages[27], it is reasonable to conclude that dTSPO depletion in fly neurons activates such pathways thus altering neuronal activity and ethanol response.

### Gender-specificity of TSPO modulation on ethanol response

The male-specific effects of TSPO inactivation were particularly striking. Previous studies have shown that male flies are more resistant to ethanol-induced sedation than females [31], which we also observed. Inactivation of dTSPO in males increased their sensitivity to ethanol, bringing their sensitivity close to that of females. Furthermore, female flies were found to have much lower dTSPO mRNA in their heads than males and knockdown of neuronal dTSPO in male heads reduced dTSPO mRNA about 20% while having no effect on the dTSPO levels of female fly heads. Thus, female flies have inherently low expression of dTSPO in their neurons and this may account for to their increased sensitivity to ethanol sedation.

In humans, men and women also exhibit different responses to acute and long-term ethanol exposure [32,33]. Men are at higher risk of AUD than women, but once AUD develops, women are more susceptible to ethanol-induced damage in multiple organs. Perhaps differences in TSPO expression contribute to human gender differences as well.

The molecular basis for the differences in dTSPO expression in flies is unknown. Male flies express a male specific splicing isoform of neuronal sex determination gene fruitless (fru), FruM. This may control the gender-specific production of neurotransmitters and neuropeptides [31]. Such a system might also regulate dTSPO expression. Additional environmental factors to which male and female animals are differentially exposed may also affect dTSPO expression.

### TSPO modulates ethanol sensitivity in adult flies

A variety of genes have been reported to control fly brain development and impact ethanol responses [5]. Since the *tspo* mutation affects all developmental stages in fly, it’s deficiency could create a developmental abnormality that alter ethanol sensitivity. However, Hematoxylin-Eosin histological staining of *tspo*-/- brains did not reveal any gross anatomical defect compared with *tspo*+/+ brains. Furthermore, by using the RU486-inducible gene switch system to knockdown dTSPO only after eclosion, we avoided any alterations in fly anatomy demonstrating that only physiological changes were important in ethanol sensitivity of adults. Hence, the ethanol sensitivity induced in male flies by the knockdown of dTSPO cannot be due to developmental alterations, but must be the product of the physiology of the adult neurons. This means that physiological modulation should be able to treat alcoholism.

The knockdown of dTSPO in neurons demonstrates that neuronal expression of TSPO is important in determining ethanol sensitivity. This neuronal action of TSPO is at variance with reports in mammals that TSPO probes co-localize primarily with glial [34,35]. That dTSPO must be expressed in neurons is not only confirmed by the current ethanol studies but also by our previous observations that systemic depletion of dTSPO protects flies from toxicity of neuronally-expressed Aβ42 [20]. Unfortunately, our current data do not indicate if the ethanol sensitivity effects of dTSPO knockdown are related to a specific group of neurons. Mammalian TSPO has been reported to function in hippocampal neurons to affect long-term potentiation and learning[36]. Also, ethanol effects have been reported for the KCNQ channel expressed in dopaminergic neurons[37] and PKA expressed in insulin-producing neurons [38].

### Possible involvement of TSPO in addiction of benzodiazepines

Benzodiazepines are widely used for treatment of anxiety, insomnia, seizures and other neural disorders, and are known to enhance the effects of GABA at the GABA_A_ receptor. However, long-term use of these drugs is controversial due to decreasing effectiveness, physical dependence, and withdrawal [39,40]. TSPO is also a target of benzodiazepines and our results suggest that benzodiazepine-derived antagonists might increase sensitivity to ethanol and decrease neurological damage [20] while benzodiazepine-derived agonists could have the opposite effects. Consequently, the TSPO may provide an important drug target for treatment of drug abuse and alcoholism [36] which could be conveniently investigated with the current system.

### Conclusion

Our data demonstrate that the mitochondrial TSPO protein, also known as the peripheral benzodiazepine receptor, is important in determining both ethanol sensitivity and the development of ethanol tolerance. Given the existing of a broad range of benzodiazepine analogues, these compounds may provide a novel approach for treating AUDs.

## Materials and Methods

### Fly stocks and culture

Flies were raised on standard cornmeal medium in narrow (25x95mm) vials at 25°C, with 12 hours/12 hours light/dark cycles. The *tspo*[EY00814] strain, obtained from the Bloomington Drosophila Stock Center (Bloomington, IN, USA), has a P-element insertion in the 3' regulatory region of *tspo* gene. The UAS-dTSPO-RNAi stock was obtained from Vienna Drosophila RNAi Center (VDRC, Vienna, Austria) and contained a transgene which can be transcribed into a dsRNA that targets the dTSPO mRNA. Pan-neuronal gene switch Gal4 driver, elav-GeneSwitch, was also obtained from Bloomington Drosophila Stock Center. These strains were all backcrossed to w^1118^ (isoCJ1) background. UAS-p35 stock was kindly provided by Dr. Nancy Bonini in University of Pennsylvania. The *rempA*[e02928] strain was also obtained from Bloomington Drosophila Stock Center.

To induce gene switch, flies combining elav-GeneSwitch with UAS-dTSPO-RNAi (elav-GS/+;TSPO-IR/+) or UAS-p35 (elav-GS/+;UAS-p35) were raised in regular food with 50 μl 4 mg/ml ethanol solution of mifepristone (RU486, Sigma-Aldrich, St Louis, MO, USA) added on the surface of food in vials for 3 days. As control, the flies were raised in regular food with 50 μl ethanol. The food vials were changed every 24 hours. In N-Acetyl-L-Cystein (NAC) experiments, 20 μl 500 mM NAC (Sigma-Aldrich) water solution or pure water was pre-mixed with 50 μl RU486 solution or ethanol solvent and then added on the surface of food in vials. In NAC experiments, the drug feeding was extended to 5 days.

### Ethanol sedation assay

Flies at 4–7 dae age were used for all sedation, recovery and tolerance assays, except for the NAC experiment where 6–9 dae flies were used. In the aging experiments, 19–22 or 34–37 dae flies were studied. Flies were sorted under CO_2_ and loaded into empty narrow (25×95mm) vials. Ten flies were loaded into a vial as a single trial and allowed to recover for at least 2 hours before use. Ethanol solutions of 34%, 44%, 54% (weight/vol) were made by mixing absolute ethanol (Sigma-Aldrich, catalog number E7023, for molecular biology) and ultrapure distilled water (Gibco, Grand Island, NY, USA) at the ratio (vol/vol) of 4:6, 5:5, and 6:4, respectively. For each vial, regular cotton clog was replaced with clog added with 1 ml ethanol solution at the vial-side surface. Recording to number of sedated flies started immediately. The interval for recording was 2 or 5 minutes.

To monitor the recovery, the ethanol-containing clog was replaced with regular clog immediately after all flies were sedated. The number of flies remaining sedated was counted every 2 or 5 minutes. For tolerance assays, the flies were transferred into regular food vial with regular clog after all flies were sedated. Four hours later, the flies were transferred back into empty vial and the recording for sedation was performed as same as in naïve flies.

### Internal ethanol content assay

Internal ethanol content was measured with Abcam Ethanol Assay Kit (Ab65343, Abcam, Cambridge, MA, USA). In brief, twenty flies at 4–7 dae age were CO_2_ anesthetized and loaded into empty narrow vial. After 2 hours recovery, flies were exposed to ethanol vapor from cotton clog soaked with 44% ethanol solution for 6 minutes when >90% flies were inactive. Then flies were quickly frozen in liquid nitrogen and homogenized in lysis buffer provided by kit and then centrifuged for 14000 g for 10 min in 4°C. The diluted sample together with standard ethanol samples were incubated in 96-well plate wells with ethanol oxidation reaction mix to produce H_2_O_2_ which further reacts with the probe in the mix to generate color. The absorbance at 570 nm was measured with a plate reader (SpectraMax Paradigm, Molecular Devices, Sunnyvale, CA, USA). The content of ethanol was calculated based on the standard curve, and finally normalized by the total protein concentration measured by the Bradford method.

### Quantitative RT-PCR

Total RNA was extracted from bodies of 20–40 flies or 100 fly heads using RNeasy Mini Kit (Qiagen, Valencia, CA, USA). The RNA was converted to cDNA using oligo(dT)15 (Invitrogen, Grand Island, NY, USA) and SuperScript II reverse transcriptase (Invitrogen). After reverse transcription, PCR reactions were performed using a ViiA7 Real-Time PCR System (Applied Biosystems, Grand Island, NY, USA) with SYBR Green Master Mix (Applied Biosystems) and primers for rp49 (forward, 5- gctaagctgtcgcacaaatg -3, and reverse, 5- ccaggaacttcttgaatccg -3) or dTSPO (forward, 5- ctcttcgtaccctacgtcgc -3, and reverse, 5- ctggttcgataggtcggaaa -3). The PCR protocol involved denaturation at 95°C for 15 seconds and combined annealing and extension at 60°C for 1 min over 40 cycles. The melting curve was generated after these cycles to ensure that the amplification in each reaction was specific.

### Caspase 3/7 activity

Isolated fly heads or whole bodies were homogenized in Homogenization Buffer (225 mM mannitol, 75 mM sucrose, 10 mM MOPS, 1 mM EGTA, pH 7.2) on ice, then centrifuged at 300 g for 5 min. The supernatant was collected and added in 96-well plate wells together with an equal volume of reaction buffer (ApoONE kit, Promega, Fitchburg, WI, USA). The plate was shaken gently for 5 min, and then incubated in dark for 15 hours in room temperature. Fluorescence was measured with a plate reader (SpectraMax Paradigm, Molecular Devices) with the excitation at 499 nm and emission at 521 nm. The fluorescent values were normalized by total protein concentration measured by the Bradford method, and the relative activity was calculated based on the ratio of normalized fluorescent signals between samples.

### Hydrogen peroxide content

Isolated fly heads were homogenized in Homogenization Buffer on ice. The samples were then centrifuged at 14000 g for 10 min in 4°C to collect the supernatant. The standard reaction solution containing 0.1 mM Amplex UltraRed, Invitrogen and 0.2 U/L horseradish peroxidase (Thermo Scientific, Pittsburgh, PA, USA) diluted in Homogenization Buffer was placed in 96-well plate wells. Then the fly extract samples or standard H_2_O_2_ samples were added to the plates and incubated for 15 min in the dark at room temperature. The fluorescence was measured with a plate reader (SpectraMax Paradigm, Molecular Devices) with the excitation at 530 nm and emission at 590 nm. The H_2_O_2_ content was calculated based on standard and normalized to the total protein concentration measured by the Bradford method.

### Histological staining

Fly heads were fixed in standard Bouin's Fixative, embed in paraffin blocks, and sectioned at a thickness of 6 μm. Sections were placed on slides, stained with haematoxylin and eosin (Vector), and examined by bright-field microscopy.

## Supporting Information

S1 Fig Equal ethanol sedation sensitivity in male flies when ethanol vapor was given bottom-up or top-down. Wild type (*tspo*+/+) male flies were tested for ethanol sedation in vials with 44% ethanol solution-soaked cotton clog closed at top (top-down vapor) or bottom (bottom-up vapor). N = 7 vials tested. Data presented as mean ± SEM.
(TIFF)

S2 Fig Comparable ethanol sedation sensitivity of *rempA*-/- and +/+ male flies. Sedation sensitivity to acute exposure of 34% ethanol vapor was comparable in *rempA*-/- and wild type flies. *tspo*-/- male flies exhibited increased sedation sensitivity under same condition. Data presented as mean ± SEM. N = 11.
(TIFF)

S3 Fig Similar gross anatomical appearance of the brains of tspo-/- and +/+ flies. Head sections of male adult flies (5–8 dae) were stained with haematoxylin and eosin.
(TIFF)

S4 Fig Increased ethanol sensitivity in male neuronal dTSPO knockdown flies. Gene switch was induced as in Fig 2. (A-C) Male elav-GS/+; TSPO-IR/+ flies with and without RU486. (A) Sensitivity to acute sedation from 34% ethanol vapor, n = 4 vials tested. (B) Sensitivity to acute sedation from 54% ethanol vapor, n = 12. (C) Delayed recovery following sedation with 54% ethanol vapor, n = 6. (D) Internal ethanol content in whole bodies of elav-GS/+; TSPO-IR flies with or without RU486, n = 3 groups of flies tested. Data presented as mean ± SEM.
(TIFF)

S5 Fig Comparable ethanol sensitivity in female neuronal dTSPO knockdown flies to control flies. Female elav-GS/+; TSPO-IR/+ flies with or without RU486. Gene switch was induced as in Fig 2. (A) Sensitivity to acute sedation from 34% ethanol vapor, n = 4, number of vials tested. (B) Sensitivity to acute sedation from 44% ethanol vapor, n = 8. (C) Slight delay in recovery following sedation with 44% ethanol vapor, n = 8. Data presented as mean ± SEM.
(TIFF)

S6 Fig Aging-associated increase in sensitivity to ethanol sedation is attenuated in male *tspo*-/- flies. Young (4–7 dae), mid-age (19–22 dae) and old (34–37 dae) male *tspo* +/+ and *tspo*-/- flies were exposed to (A) 44% ethanol vapor or (B) 54% ethanol vapor. The differential increased resistance seen in *tspo* +/+ flies relative to *tspo*-/- flies is lost when the *tspo* +/+ flies exceed 20 dae. N = 2~5 vials tested for each trace. Data presented as mean ± SEM.
(TIFF)

S7 Fig Ethanol tolerance is not altered in female *tspo*-/- flies compared with *tspo*+/+ flies. Female *tspo*-/- and *tspo* +/+ flies showed similar increased resistance to first and second exposure to 44% ethanol vapor indicating that a systemic lack of dTSPO does not show the same loss of tolerance as seen in male dTSPO deficient flies. First exposure, n = 10 vials tested; second exposure, n = 4. Data presented as mean ± SEM.
(TIFF)
