# Supplementary figures and images for: TSPO, a Mitochondrial Outer Membrane Protein, Controls Ethanol-Related Behaviors in Drosophila
Source: PLoS Genet. 2015 Aug 4;11(8):e1005366. doi: 10.1371/journal.pgen.1005366 (PMC4524697; doi:10.1371/journal.pgen.1005366)

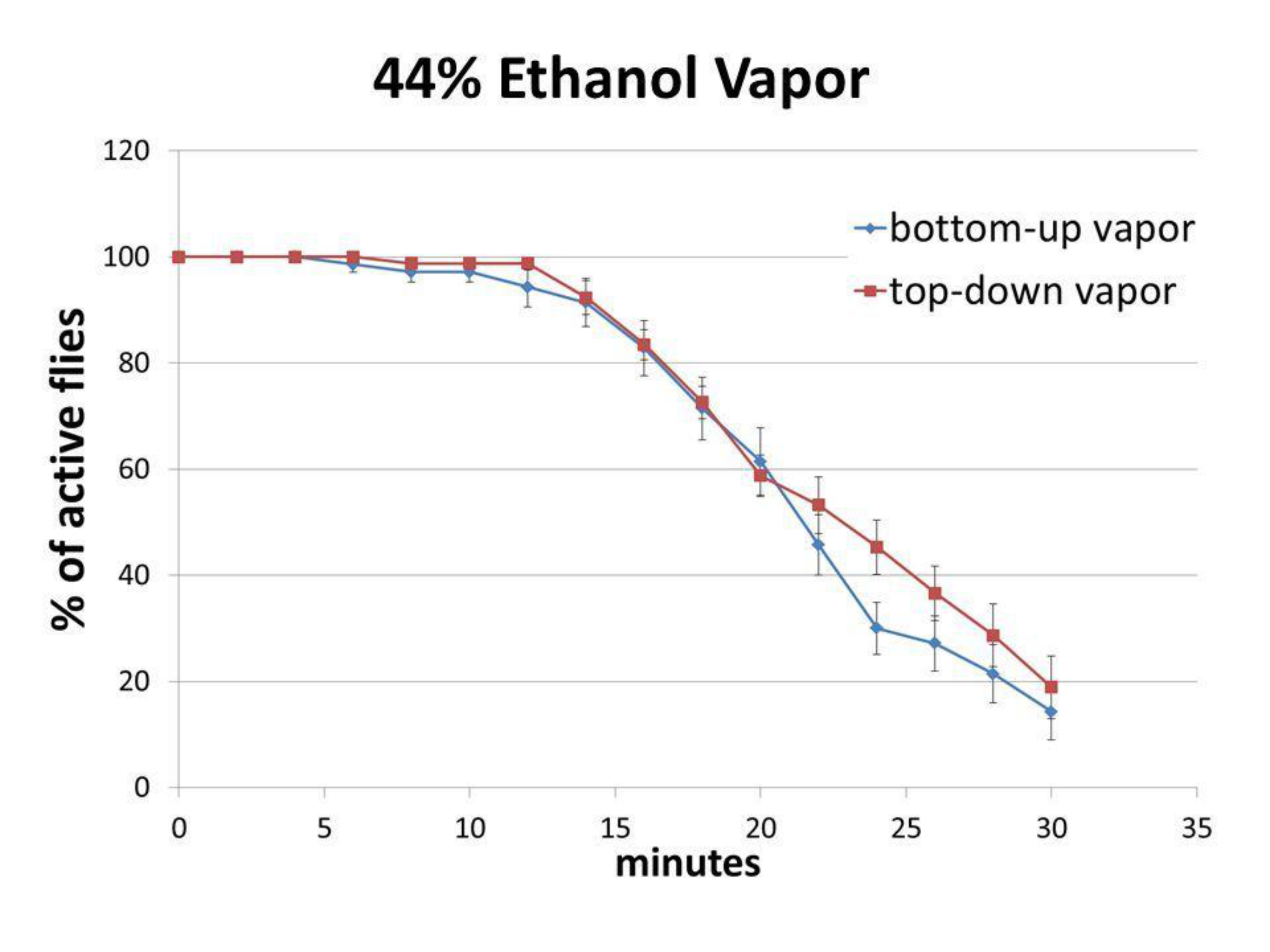

Supplement: S1 Fig — Wild type (tspo+/+) male flies were tested for ethanol sedation in vials with 44% ethanol solution-soaked cotton clog closed at top (top-down vapor) or bottom (bottom-up vapor). N = 7 vials tested. Data presented as mean ± SEM. (TIFF) [file pgen.1005366.s001.tiff]

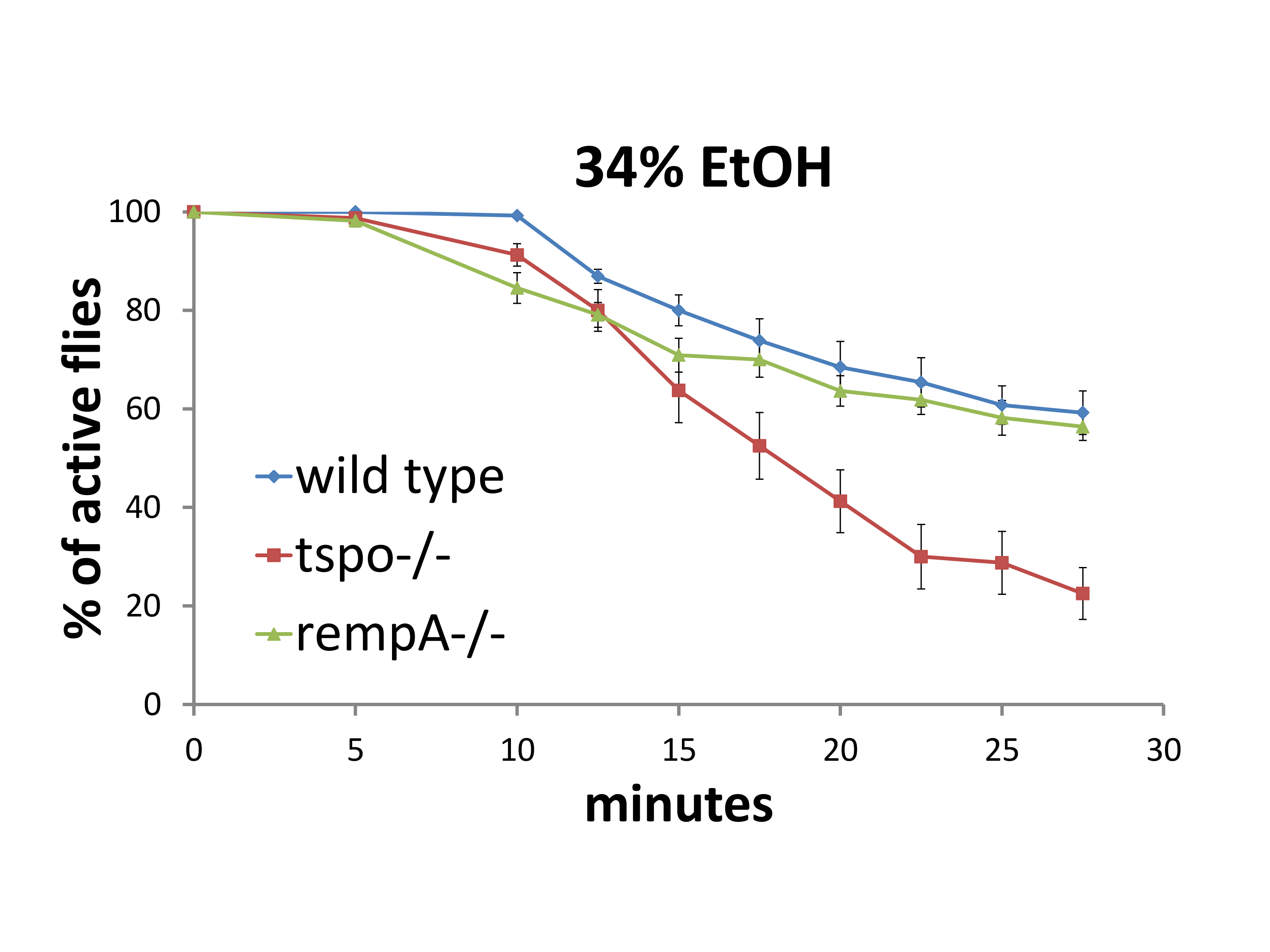

Supplement: S2 Fig — Sedation sensitivity to acute exposure of 34% ethanol vapor was comparable in rempA-/- and wild type flies. tspo-/- male flies exhibited increased sedation sensitivity under same condition. Data presented as mean ± SEM. N = 11. (TIFF) [file pgen.1005366.s002.tiff]

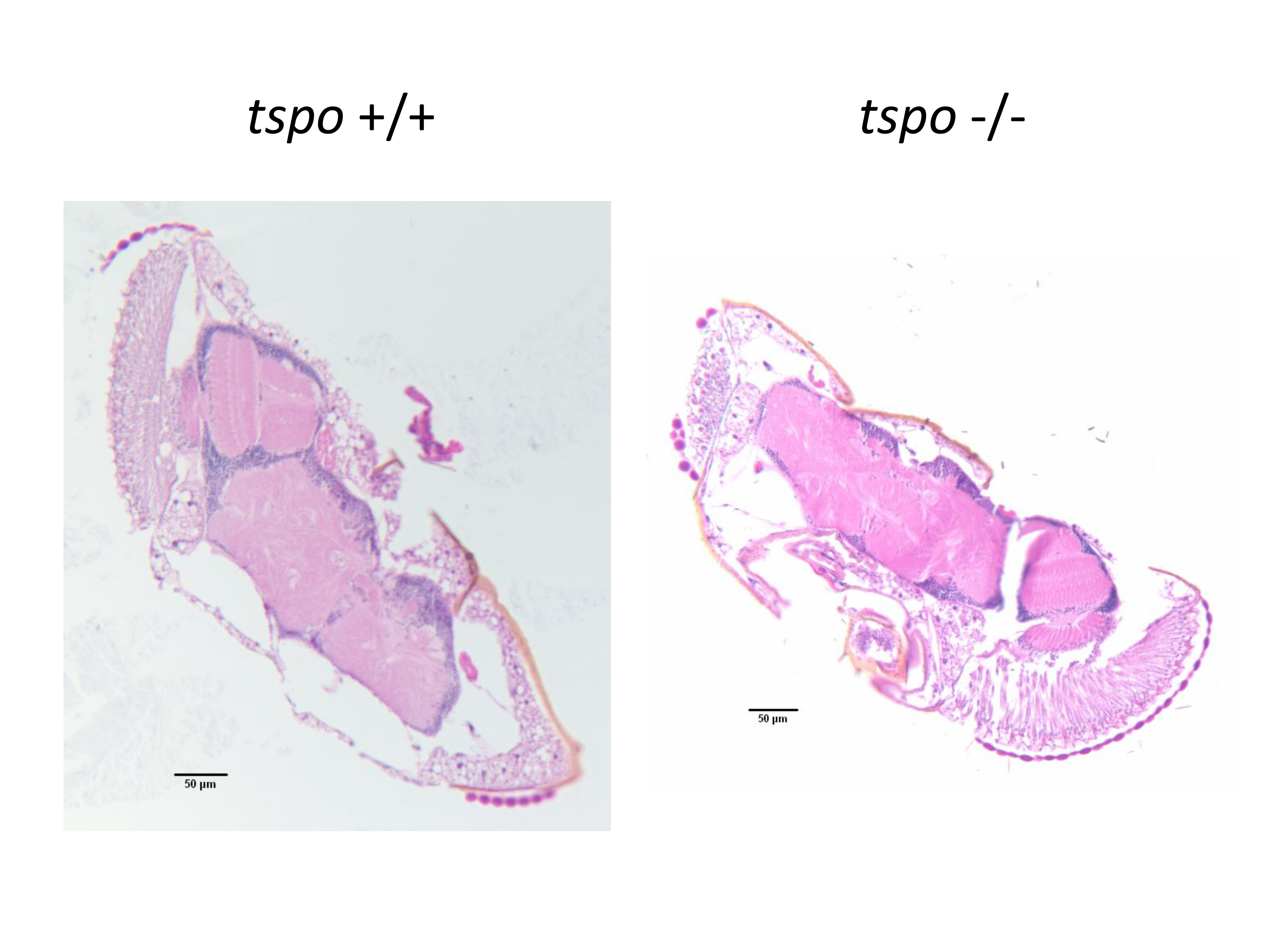

Supplement: S3 Fig — Head sections of male adult flies (5–8 dae) were stained with haematoxylin and eosin. (TIFF) [file pgen.1005366.s003.tiff]

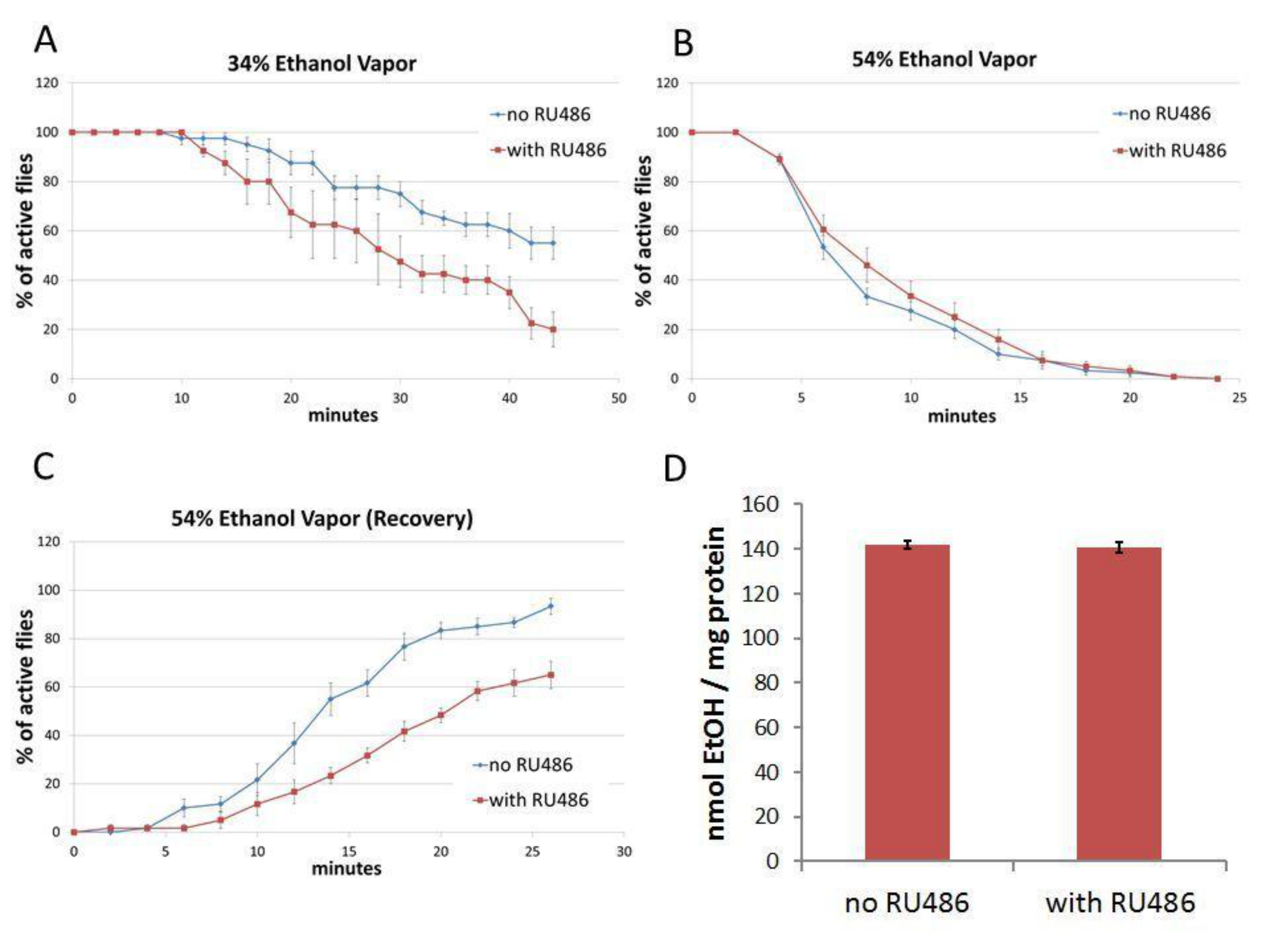

Supplement: S4 Fig — Gene switch was induced as in Fig 2. (A-C) Male elav-GS/+; TSPO-IR/+ flies with and without RU486. (A) Sensitivity to acute sedation from 34% ethanol vapor, n = 4 vials tested. (B) Sensitivity to acute sedation from 54% ethanol vapor, n = 12. (C) Delayed recovery following sedation with 54% ethanol vapor, n = 6. (D) Internal ethanol content in whole bodies of elav-GS/+; TSPO-IR flies with or without RU486, n = 3 groups of flies tested. Data presented as mean ± SEM. (TIFF) [file pgen.1005366.s004.tiff]

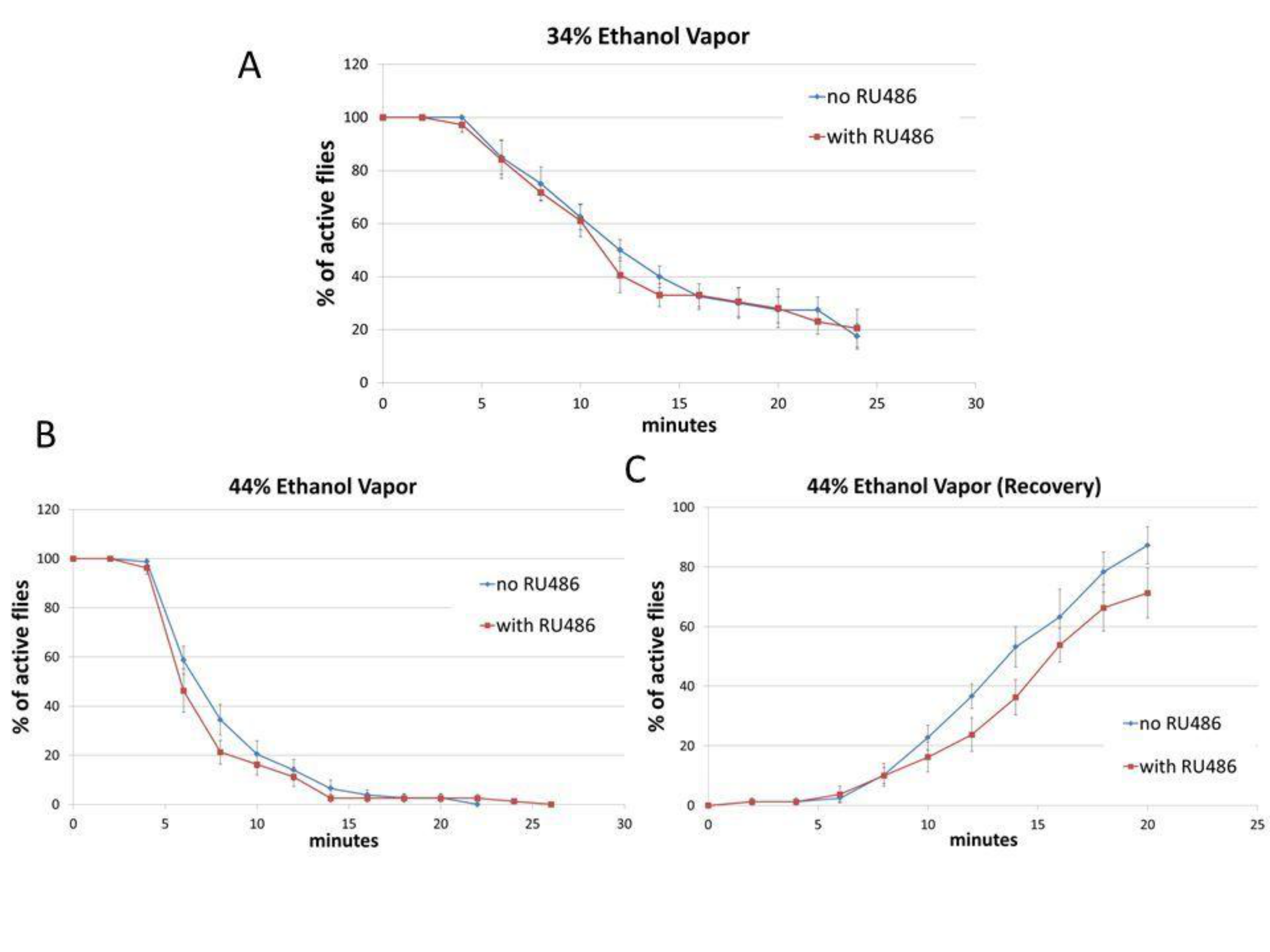

Supplement: S5 Fig — Female elav-GS/+; TSPO-IR/+ flies with or without RU486. Gene switch was induced as in Fig 2. (A) Sensitivity to acute sedation from 34% ethanol vapor, n = 4, number of vials tested. (B) Sensitivity to acute sedation from 44% ethanol vapor, n = 8. (C) Slight delay in recovery following sedation with 44% ethanol vapor, n = 8. Data presented as mean ± SEM. (TIFF) [file pgen.1005366.s005.tiff]

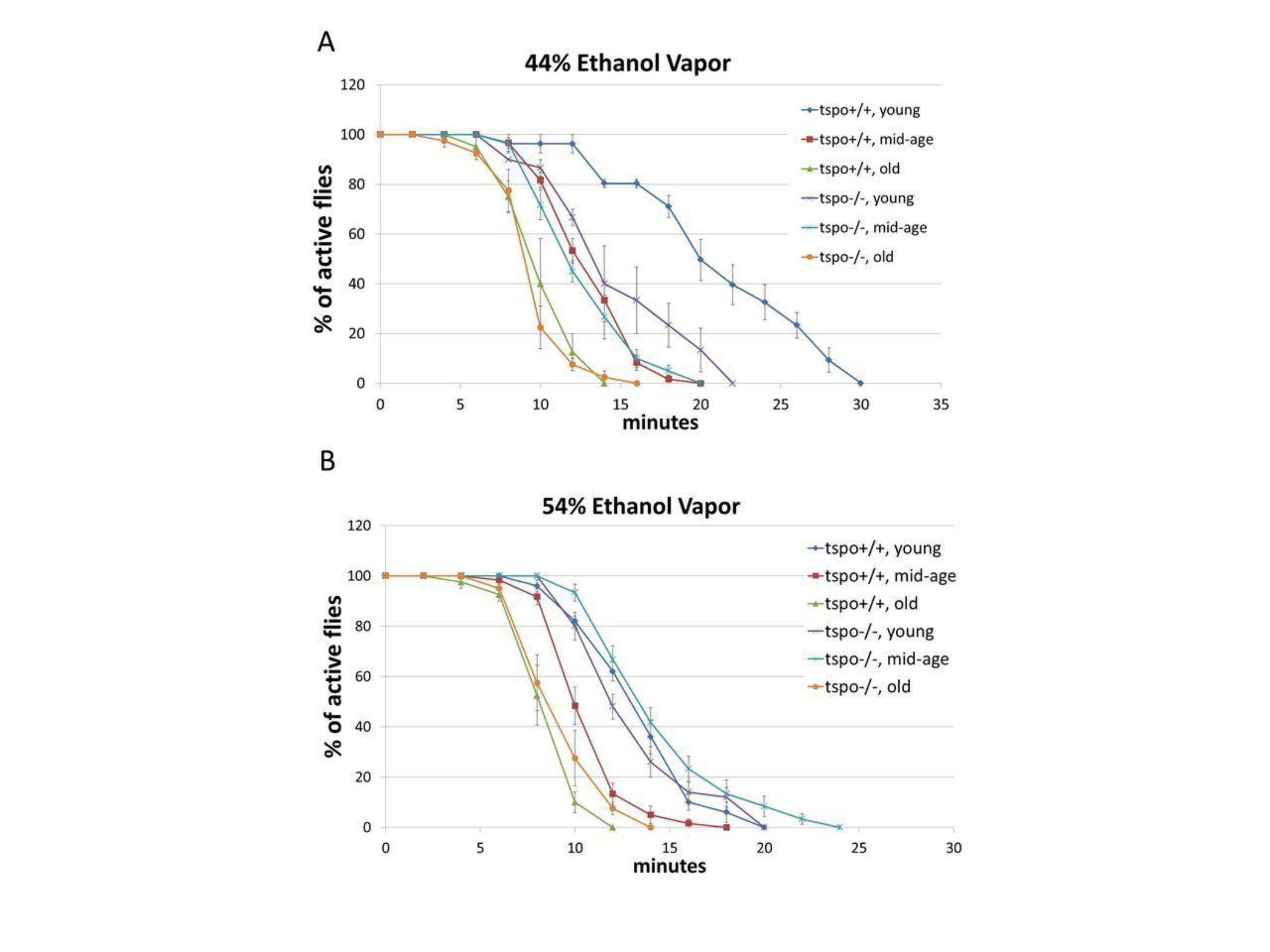

Supplement: S6 Fig — Young (4–7 dae), mid-age (19–22 dae) and old (34–37 dae) male tspo +/+ and tspo-/- flies were exposed to (A) 44% ethanol vapor or (B) 54% ethanol vapor. The differential increased resistance seen in tspo +/+ flies relative to tspo-/- flies is lost when the tspo +/+ flies exceed 20 dae. N = 2~5 vials tested for each trace. Data presented as mean ± SEM. (TIFF) [file pgen.1005366.s006.tiff]

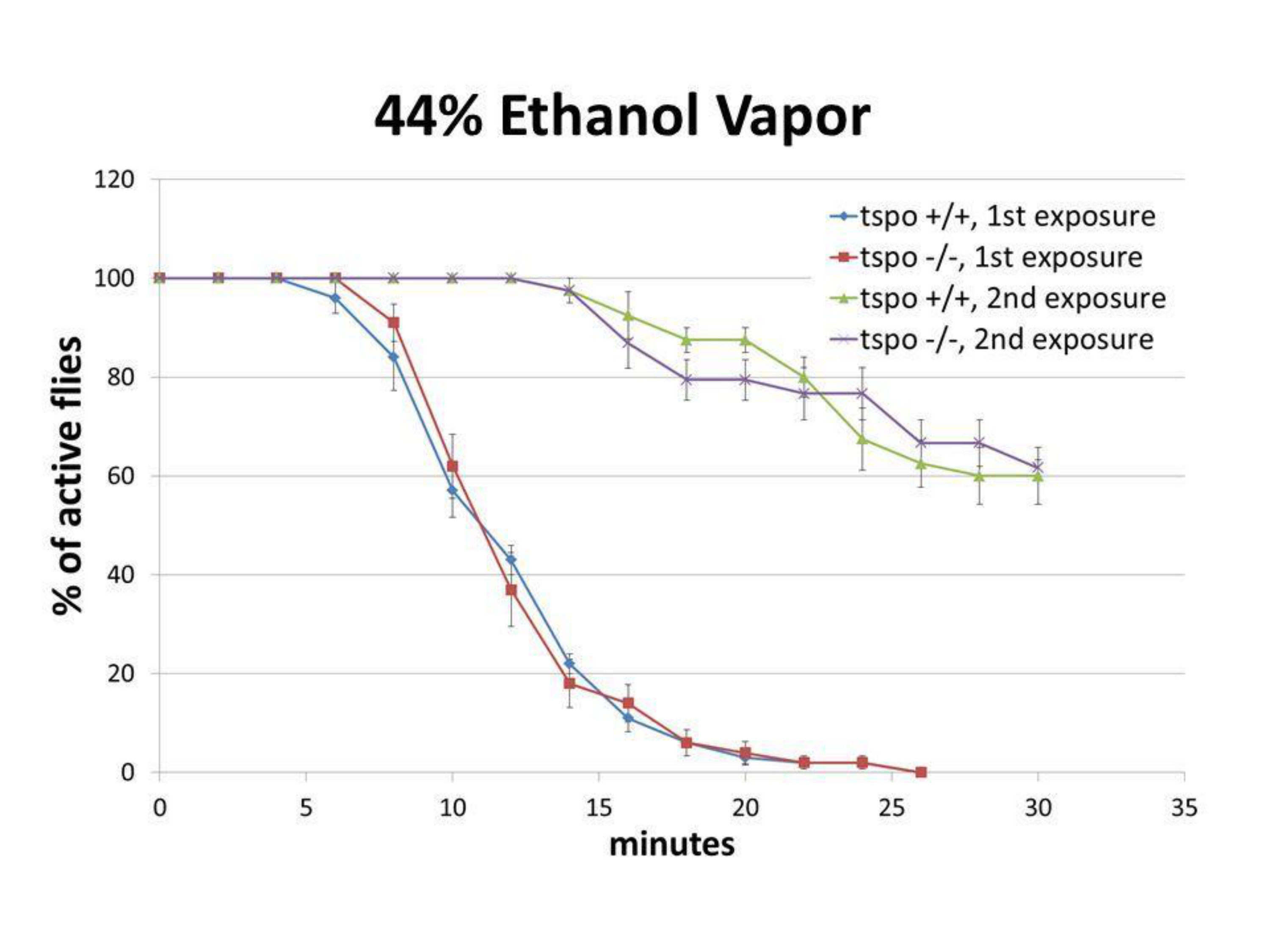

Supplement: S7 Fig — Female tspo-/- and tspo +/+ flies showed similar increased resistance to first and second exposure to 44% ethanol vapor indicating that a systemic lack of dTSPO does not show the same loss of tolerance as seen in male dTSPO deficient flies. First exposure, n = 10 vials tested; second exposure, n = 4. Data presented as mean ± SEM. (TIFF) [file pgen.1005366.s007.tiff]
